# Interstitial Lung Disease Associated Acute Respiratory Failure Requiring Invasive Mechanical Ventilation: A Retrospective Analysis

**DOI:** 10.2174/1874306402014010067

**Published:** 2020-12-18

**Authors:** Cyrus A. Vahdatpour, Alexander Pichler, Harold I. Palevsky, Michael J. Kallan, Namrata B. Patel, Paul A. Kinniry

**Affiliations:** 1 Division of Pulmonary, Critical Care, and Sleep Medicine, University of Florida, Gainesville, Florida; 2 Department of Internal Medicine, Medical University of Vienna, Vienna, Austria; 3 Pulmonary, Allergy, and Critical Care Division, Department of Medicine, Penn Presbyterian Medical Center, Philadelphia, Pennsylvania; 4 Perelman School of Medicine, University of Pennsylvania, Philadelphia, Pennsylvania; 5 Department of Biostatistics, Epidemiology, and Informatics, Perelman School of Medicine, University of Pennsylvania, Philadelphia, Pennsylvania; 6 Pulmonary, Allergy, and Critical Care Division, Department of Medicine, Hospital of the University of Pennsylvania, Philadelphia, Pennsylvania; 7 Pulmonary, Allergy, and Critical Care Division, Department of Medicine, Pennsylvania Hospital, Philadelphia, Pennsylvania

**Keywords:** Intensive care, Acute respiratory failure, Interstitial lung disease, Mechanical ventilation, High dose steroids, Lung transplant

## Abstract

**Background:**

Interstitial Lung Disease [ILD] patients requiring Invasive Mechanical Ventilation [IMV] for Acute Respiratory Failure [ARF] are known to have a poor prognosis. Few studies have investigated determinants of outcomes and the utility of trialing Non-Invasive Positive Pressure Ventilation [NIPPV] prior to IMV to see if there are any effect[s] on mortality or morbidity.

**Methods:**

A retrospective study was designed using patients at four different intensive care units within one health care system. The primary objective was to determine if there are differences in outcomes for in-hospital and one-year mortality between patients who undergo NIPPV prior to IMV and those who receive only IMV. A secondary objective was to identify potential determinants of outcomes.

**Results:**

Out of 54 ILD patients with ARF treated with IMV, 20 (37.0%) survived until hospital discharge and 10 (18.5%) were alive at one-year. There was no significant mortality difference between patients trialed on NIPPV prior to IMV and those receiving only IMV. Several key determinants of outcomes were identified with higher mortality, including higher ventilatory support, idiopathic pulmonary fibrosis (IPF) subtype, high dose steroids, use of vasopressors, supraventricular tachycardias (SVTs), and higher body mass index.

**Conclusion:**

Considering that patients trialed on NIPPV prior to IMV were associated with no mortality disadvantage to patients treated with only IMV, trialing patients on NIPPV may identify responders and avoid complications associated with IMV. Increased ventilator support, need of vasopressors, SVTs, and high dose steroids reflect higher mortality and palliative care involvement should be considered as early as possible if a lung transplant is not an option.

## 1 INTRODUCTION

Interstitial Lung Disease [ILD] patients can be subject to episodes of Acute Respiratory Failure [ARF] and rapid decline during their disease course. Patients with ILD experiencing ARF have known poor outcomes once Invasive Mechanical Ventilation [IMV] is initiated. Retrospective studies have reported in-hospital mortality, ranging from 51-100% in ILD patients with ARF requiring ICU level of care [1-6]. IMV may be required in ILD-Associated ARF [ILD-ARF], although its benefits are unclear, unless used as a bridge to Lung Transplantation [LTx].

ARF from an ILD exacerbation is defined as a subjective worsening of dyspnea within the month prior to presentation; new ground glass opacities or consolidation by chest imaging; hypoxemia with >10mmHg decline in PaO2; and no evidence of PE, CHF, lung infection, or pneumothorax [7, 8]. This definition has been modified for the acute exacerbation of Idiopathic Pulmonary Fibrosis [IPF] to now include pulmonary infection as an etiology, but this modification has not been incorporated into the other ILD subtypes [9].

IMV can initiate and exacerbate lung injury, termed as Ventilator-Induced Lung Injury [VILI], increasing mortality, and morbidity [6]. Non-Invasive Positive Pressure Ventilation [NIPPV] may offer some benefits of IMV, by improving oxygenation and reducing the work of breathing, and minimizing the risk for VILI. The effectiveness of NIPPV in avoiding IMV in patients with ILD-ARF has not been well studied. Whether or not trialing NIPPV, and potentially delaying IMV, increases mortality is also unclear.

There are no guidelines on how to select ILD-ARF patients to place on IMV. Gungor *et al*. [2013] proposed that physicians should be guarded about the use of IMV in ILD-ARF patients that are not suitable for Lung Transplantation [LTx], especially in patients requiring continuous NIPPV [5]. There are several studies looking at IPF patients who require IMV, revealing high in-hospital mortality. Although IPF is the most common idiopathic interstitial pneumonia, it represents a fraction of patients with ILD who have ARF [7]. The outcomes of ILD patients, as a whole, have been less frequently reported. While there are individuals who survive IMV, there is limited data to differentiate these individuals from those who do not. As a result, this limits the ability to have informed goals of care discussions for critically ill patients.

The primary objective of this study was to investigate mortality outcomes between two cohorts of ILD-ARF patients [1]: those who are trialed on NIPPV prior to receiving IMV and [2] those receiving only IMV. The secondary objective was to identify the determinants of outcomes within the entire study population and within each stated cohort.

## 2 METHODS

### 2.1 Study Population

From January 2014 to October 2018, 54 patients with ILD-ARF underwent IMV in 4 different hospitals within the University of Pennsylvania Health System. Patients were identified by using the International Classification of Diseases [ICD] codes to search within the institution’s Electronic Medical Record [EMR]. Institutional Review Board [IRB] approval from the University of Pennsylvania was obtained prior to the review of medical records.

Patient’s records were reviewed if they were previously diagnosed with ILD and if ARF was experienced during their admission. Only the first presentation of ARF requiring IMV was used in this study in cases of patients with repeat admissions for ARF. Their ARF had to require any form of Positive Pressure Ventilation [PPV] initially and ultimately required IMV for 24 hours [6].

ILD diagnosis based on the American Thoracic Society criteria was limited in assessment based on EMR review. To improve the accuracy of the ILD diagnosis, major and minor criteria were created for patient eligibility. Major criteria included: an available CT scan read by a radiologist that was suggestive of ILD and either co-existing restrictive PFTs or pulmonologist documentation that confirmed ILD as a clinical diagnosis; available tissue biopsy confirming diagnosis; or documented proof of lung transplant screening due to an ILD. Minor criteria included: either stated ILD in prior and/or current physician encounter, a CT scan referencing ILD, a Pulmonary Function Test [PFT] with restrictive profile, an ICD code for ILD in the EMR, and pulmonary provider documentation noting ILD in current encounter. A patient required either one of the major criteria or three minor criteria to be included in this study. Refer to Figure S1 in the supplemental materials file for a depiction of major and minor criteria.

Patients were excluded if they had a chronic tracheostomy, were surgical patients experiencing ARF within 48 hours after being extubated for a surgical procedure, had no ARF during their encounter, were intubated for reasons that were unrelated to ARF, or had a history of having undergone LTx.

We defined ARF based on the British Medical Journal's best practice guidelines [10]. Once a patient met the criteria of ARF, we required evidence of supplemental oxygenation use that ultimately needed to be escalated to PPV for respiratory support. For patients already on home oxygen, we defined ARF as an increased oxygen requirement from their home requirement. All patients included in this study eventually required IMV support for greater than 24 hours for ARF.

PH diagnosis was based on prior ICD coding or defined in patients with recent echocardiography report within 6 months of encounter or during the encounter with an sPAP ≥ 45mmHg [11].

Pulse dose steroids were defined as 1g of methylprednisolone for a minimum of 3 days [12]. Stress dose steroids were defined as a documented administration at no more than 300mg of hydrocortisone [or equivalent] in a 24 hour time period [13, 14] for the duration of shock physiology or death. Observed stress dose steroid regimens were 50mg of hydrocortisone every 6 hours or 100mg of hydrocortisone every 8 hours.

### 2.2 Data Collection

Primary study outcomes were survival until hospital discharge and at one-year, in patients trialed on NIPPV prior to IMV and in patients only treated with IMV. As a secondary outcome, we investigated the possible determinants of the primary outcomes, including demographic data, baseline patient characteristics, ILD subtype, ventilator settings, echo cardiography, and ICU level interventions. We also stratified patients based on the presence or absence of pulmonary hypertension to see if this had any impact on primary or secondary outcomes. Cardiothoracic imaging, PFTs, lung histopathology, lab values, ventilator settings, and patient history were collected from EMR. Mechanical ventilation settings were recorded at the end of a 24-hour time interval from its first initiation during their hospital admission. For patients transferred from another institution, mechanical ventilator settings were recorded from [1] their documented ventilator settings prior to transfer that was closest to the 24 hour time interval from its first initiation, or [2] ventilator settings on arrival to our institution, provided they were on IMV for more than 24 hours and had no ventilator settings available prior to transfer.

### 2.3 Statistical Analysis

Results are presented as a median and interquartile range [IQR] for quantitative variables and frequencies and percentages for qualitative variables. Fisher’s exact tests were used for binominal variables and variables with more than two labels, as appropriate. One-year survival was determined from the date of onset from ARF. The Kaplan-Meier survival curves were plotted for survival data with statistical evaluation through Mantel-Cox log-rank statistics. The threshold for statistical significance was p < 0.05.

## 3 RESULTS

**Fig. (1):**
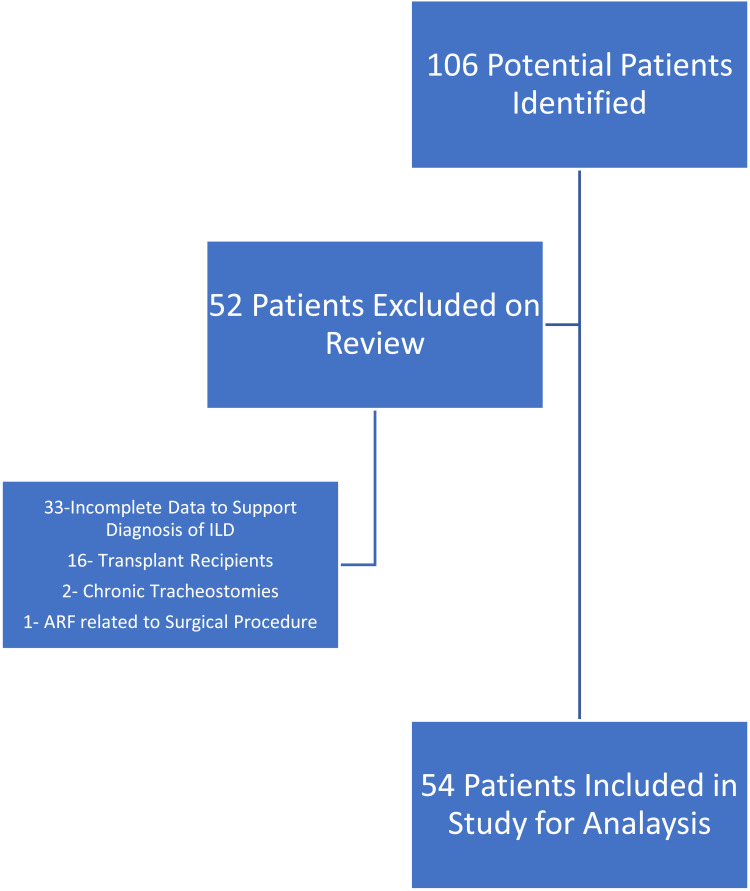
Graphical representation of patients meeting inclusion criteria.

We identified 106 potential ILD patients who were admitted to our health system between January 2014 and October 2018, with ARF and who required IMV. Fifty-two patients were excluded: 33 patients did not have enough data to support the ILD diagnosis; 16 patients were previously LTx recipients; 2 patients had chronic tracheostomies, and 1 patient had experienced a surgical related ARF. The remaining 54 patients fulfilled the inclusion criteria and were included in the analysis (Fig. **1**).

### 3.1 General Baseline Characteristics

Tables **1** and **2** provide a detailed description of patient characteristics. The mean age of patients was 65 years and males represented 55.6% of the total cohort. An ILD diagnosis for greater than 1 year was seen in 55.6% of patients. Connective Tissue Disease [CTD] was the most commonly identified cause of the ILD diagnosis, representing 31.5% of the patients and Idiopathic Pulmonary Fibrosis [IPF] was the second most common diagnosis, representing 14.8% of the patients. Chronic steroid use was found in 56.0% of the patients and 40.0% of the patients were on other chronic immunosuppressants. 55.6% of patients were transferred from another institution. Home oxygen supplementation was used in 46.3% of patients.

### 3.2 Admission Characteristics

The average duration of hospital admission was 22 days and the average duration of ICU admission was 18 days. The average duration on IMV was 13.6 days and the average duration on any form of PPV was 14.3 days. Pulse dose steroids were administered to 25.0% of patients and 21.0% were treated with stress dose steroids. Super Ventricular Tachycardia [SVT] complicated 90.0% of patients during their hospital course. Vasopressors were used in 85.0% of patients during their hospital course.

Assist control/volume control ventilation was the most commonly used ventilation mode. Norepinephrine was the most common first vasopressor utilized in patients with shock physiology. Tracheostomy was performed in 18.5% of patients, which was equally balanced in both cohorts. LTx was documented to be considered in 11 [22.0%] patients, 2 of whom received LTx and were both alive at one year. 6 patients were supported with ECMO: two died, two were weaned off and ultimately discharged, and two were the recipients of LTx. Palliative care consultation was performed in 48.8% of patients.

Additional patient characteristics are referenced in Supplemental Tables S1-3 within the results section of the supplemental materials file. Table S1 highlights ILD subtypes of our patient cohort. Table S2 depicts the etiology of ARF within our cohort. Table S3 characterizes ICU and Ventilator data for our cohort.

### 3.3 Mortality and Determinants

Of the 54 ILD patients with ARF treated with IMV, 20 [37.0%] survived to hospital discharge and 10 [18.5%] were alive at one-year (Table **3**). No mortality difference was observed in patients who were trialed on NIPPV prior to IMV versus those only treated with IMV. Significant increases in in-hospital mortality were found in patients with a higher BMI, the IPF subtype, vasopressor use, and stress or pulse steroids administration. Patients already on supplemental oxygenation at home and with known ILD diagnosis of greater than one year were found to have lower in-patient mortality. Analysis of mechanical ventilation parameters impacting in-hospital mortality is demonstrated in Table **4**. Ventilator parameters with increased PEEP and FiO2 settings and higher documented average airway pressures were associated with higher in-hospital mortality. Fig. (**2**) demonstrates the significant mortality differences between these ventilator parameters using the day of intubation as the starting point.

Significantly increased one-year mortality was found in patients with the IPF subtype, no past medical history of CTD, presence of SVT, and vasopressor use (Table **5**). In patients in the NIPPV prior to IMV cohort, an age greater than 65 years was associated with increased one-year mortality in comparison to those treated with only IMV. In the NIPPV prior to IMV cohort, those without pulmonary hypertension had higher one-year mortality.

When splitting the patient cohort based on the presence or absence of PH, there was no significant mortality difference [Table S6]. Additional analysis of determinants relating to in-hospital and one year mortality are represented in the Tables S4 and S5 within the supplemental materials file.

## 4 DISCUSSION

This retrospective study describes the clinical course, ICU management, and outcome of 54 ILD patients requiring IMV for ARF at a tertiary-referral institution. Both in-hospital and one -year mortality was high, and no difference was found in the primary outcome. Higher ventilator support requirements, vasopressor use, high dose steroids, and the IPF subtype were associated with worse in-hospital mortality. The IPF subtype, ILD exacerbation as a cause of ARF, no past history of CTDs, presence of SVT, vasopressor use were independent predictors of one-year mortality.

One prior study has described the in-hospital and one-year mortality of ILD patients experiencing ARF, requiring IMV, as 47.0% and 41.0%, respectively [6]. Our study demonstrated a 63.0% in-hospital mortality and 81.5% one-year mortality. Differences in mortality rates are likely in part to the fact that our study had a large percentage of patients that were transferred from an outside hospital, which may reflect patients with worse prognosis having failed to respond to initial medical care prior to transfer. Additionally, a sample size of 54 patients is smaller than the study by Fernandez-Perez, who included 94 patients [6]. NIPPV trialed prior to IMV has been shown to have higher mortality in the study by Fernandez-Perez [6]. This difference may also be subject to sample size differences and more studies are needed to investigate these findings within the ILD cohort. Another study, not specific to the ILD cohort, has also suggested that patients receiving a NIPPV trial, prior to IMV, have worse clinical outcomes [15]. More patients were trialed on NIPPV for ARF [91.2%] prior to IMV in comparison to IMV only [62.7%]. Complications experienced that were significant included hypotension, desaturation, and aspiration. Our study required ILD patients to have the diagnosis of ARF as the reason for IMV to meet inclusion criteria, which is a critical difference. Mosier et al. also suggested increased mortality when a complication did occur [15]. Our study found no significant difference in in-hospital mortality and one-year mortality, in general, between the two compared cohorts. Due to differences in patient cohorts, this finding is difficult to compare with ours. Patients trialed on NIPPV prior to IMV is not a standardized intervention and there is no recognized definition of what time duration of NIPPV trial is needed before it can be considered as failure. Our study did suggest that the age of 65 years or older had worse one-year mortality in patients trialed on NIPPV prior to IMV in comparison to those undergoing only IMV.

This proposes an ethical dilemma for critical care physicians because, in general, mortality is high for ILD-ARF patients who fail a NIPPV trial and it is unclear if IMV is even worthwhile after NIPPV failure. IMV is often an intervention that has been considered futile if transplantation is not an option for patients with advanced underlying ILD; this has most commonly been discussed in IPF patients [6, 16-18]. However, NIPPV trial prior to IMV initiation may identify a subset of patients who are responders and avoid the need for IMV altogether. Thus, the effect is unclear in regard to NIPPV trials prior to IMV versus not trialing NIPPV prior to IMV. The decision to trial NIPPV prior to IMV should remain unique to the patient and the specific clinical scenario.

Fernandez-Perez described that the ventilator settings, which correlated with increased mortality were higher PEEP, lower tidal volume, and higher FiO2 [6]. They also found higher documented mean, plateau, and peak airway pressures, and lower PaO2/FiO2 ratios were associated with increased mortality. Our study supported that higher PEEP settings and higher FiO2 were associated with increased in-hospital mortality. These findings were also associated with decreased one-year survival starting from the day of intubation. This suggests that ARF-ILD patients requiring IMV are subject to increased risks of barotrauma, volutrauma, and cellular injury related to hyperoxia/free radical damage. Statistical differences in tidal volume settings, recorded peak airway pressures, and recorded PaO2/FiO2 ratios between our study and the Fernandez-Perez study are likely related to power. Additionally, lower tidal volume requirements to maintain plateau pressures suggests higher disease burden and poor compliance that is prognostically concerning. It is reasonable for physicians caring for ILD-ARF patients requiring IMV to adopt mechanical ventilator strategies from ARDS protocols that incorporate using the lowest possible tidal volumes and PEEP settings [19-21]. FiO2 should also be aggressively titrated to the lowest possible setting to avoid hyperoxic acute lung injury [22].

PH and its effect on the primary study outcomes were unclear. Our study did not support that PH impacts mortality or morbidity in ILD-ARF. Saydain *et al*. also found no difference in systolic pulmonary artery pressures between survivors and non-survivors [17]. Zafrani et al. did find that PH was a determinant for in-hospital and one-year mortality [7]. IMV in patients with severe PH should be avoided if possible due to potential complications of hemodynamic instability [23]. Right heart catheterization [RHC] remains the diagnostic modality for PH and is not commonly done in this cohort of patients. No study has evaluated IMV outcomes of ILD patients with PH diagnosed by RHC.

Zafrani et al. found that corticosteroid therapy was potentially of benefit during ILD-ARF in patients admitted to the ICU [7]. About 41% of our cohort received high dose steroids during their ICU course compared with 65% in the Zafrani et al. study. In their study, patients with less fibrosis on chest CT scan were noted to have a better response to steroids and earlier treatment with corticosteroids was associated with improved mortality. Our study found that high dose corticosteroid therapy [pulse or stress dosing] was associated with increased mortality. The reasons for differences in these findings were difficult to determine as it was unclear what the different subtypes of ARF were in their cohorts. Our cohort had ARF primarily from pneumonia/sepsis and ILD exacerbations and all of our patients underwent IMV, whereas Zafrani et al. had only 61% of patients treated with IMV during their ICU course [7]. It is difficult to compare their cohort with ours, as all our patients required IMV for respiratory support, implying that our cohort had a higher all-cause disease burden. Fernandez-Perez found that high dose corticosteroid therapy had no significant effect on ILD-ARF patients requiring IMV [6]. It remains unclear what the effect of high dose corticosteroid therapy is in patients ILD-ARF requiring IMV.

SVT, aside from sinus tachycardia, was associated with higher in-hospital [p= 0.06] and one-year mortality [p=0.037]. There is limited data on the epidemiology of AAs in ILD patients. Studies in IPF have demonstrated that SVTs are common [24, 25]. SVT can be difficult to manage in ILD-ARF because [1]: there is no standardized approach to management in this cohort, and [2] it is difficult to assess if SVT is the primary cause of ARF or if the SVT is secondary to the underlying etiology of ARF.

## LIMITATIONS

This study has several limitations that could have influenced the findings. It is limited in its retrospective study design and by low power, thus necessitating the need for further investigations to validate our discovered associations. Due to reliance on the diagnosis of ILD and ARF from ICD coding and EMR investigation, it is possible that we missed patients who were not coded properly. Additionally, EMR charting was not always reliable for data extraction and some patients had missing values when analyzing potential determinants of mortality, which could have impacted statistical significance. Our conclusions cannot be generalized to all ILD patients because our study does not include ILD-ARF patients who may not have received IMV due to either the decision for palliative care interventions, death while on NIPPV or improvement with NIPPV trial. Using cutoffs of patients on IMV for ≥24 hours may have missed ILD patients that were too ill for inclusion. Our strict inclusion criteria may have also contributed to lower power. Finally, our study is only representative of one health system and may be subject to limitations related to individualized institutional cultural practices.

## CONCLUSION

This study has several important clinical findings that support previous findings in prior studies. ILD-ARF requiring IMV has a poor prognosis. We found no difference in outcome between patients trialed on NIPPV prior to IMV versus patients treated with only IMV for their ARF. The lowest possible PEEP and FiO2 settings should be utilized in patients requiring IMV. PH was not found to influence mortality in ILD-ARF patients requiring IMV. The use of high-dose corticosteroids is unclear in ILD-ARF patients requiring IMV. The presence of SVT was associated with increased mortality and more studies are needed to define best management strategies.

## Figures and Tables

**Fig. (2):**
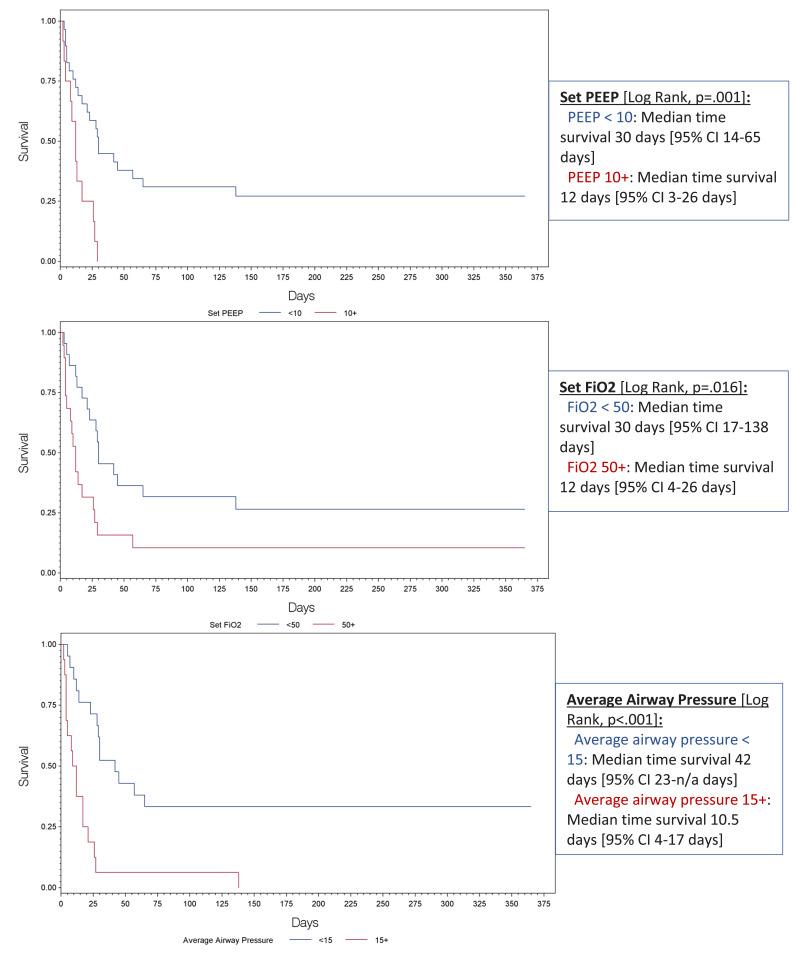
Kaplan-Meier curves for peep, fio2, average airway pressures.

**Table 1:** General patient characteristics.

-	**Total**			**NIPPV prior to IMV**				**Only IMV**		
**Variable**	**n**	**Out of**	**%**	**n**	**Out of**		**%**	**n**	**Out of**	**%**
**All Patients**	54	54	100.0%	27	27	100.0%		27	27	100.0%
**Race** Asian Black Hispanic Other Unknown White	3 13 3 3 2 30	54	5.6% 24.1% 5.6% 5.6% 3.7% 55.6%	0 7 0 2 2 16	27	0.0% 25.9% 0.0% 7.4% 7.4% 59.3%		3 6 3 1 0 14	27	11.1% 22.2% 11.1% 3.7% 0.0% 51.9%
**Female**	24	54	44.4%	13	27	48.1%		11	27	40.7%
**Smoking History** Current Never Prior Use Unknown	1 18 32 3	54	1.9% 33.3% 59.3% 5.6%	1 9 16 1	27	3.7% 33.3% 59.3% 3.7%		0 9 16 2	27	0.0% 33.3% 59.3% 7.4%
**Duration of Years with ILD** < 1 Year 1 to < 3 Years 3 to < 5 Years 5+ Years	24 12 7 11	54	44.4% 22.2% 13.0% 20.4%	11 5 5 6	27	40.7% 18.5% 18.5% 22.2%		13 7 2 5	27	48.1% 25.9% 7.4% 18.5%
**On Home Oxygen**	25	54	46.3%	15	27	55.6%		10	27	37.0%
**ILD Subtype** IPF CTDs Sarcoidosis Other	8 17 7 22	54	14.8% 31.5% 13.0% 40.7%	2 8 4 13	27	7.4% 29.6% 14.8% 48.1%		6 9 3 9	27	22.2% 33.3% 11.1% 33.3%
**Known Histological Classification** Yes No Unknown	15 34 5	54	27.8% 63.0% 9.3%	7 17 3	27	25.9% 63.0% 11.1%		8 17 2	27	29.6% 63.0% 7.4%

**Group**	**n**	**Mean [SD]**		**n**	**Mean [SD]**			**n**	**Mean [SD]**	
**Age [Years]**	54	65.2 [12.3]		27	65.3 [11.8]			27	65.0 [13.0]	
**BMI**	54	27.2 [6.2]		27	28.1 [5.9]			27	26.4 [6.5]	
**TLC**	21	59.7 [17.2]		12	60.8 [20.9]			9	58.1 [11.7]	
**FVC**	34	52.4 [17.0]		16	51.6 [15.4]			18	53.0 [18.7]	
**FEV1/FVC**	34	91.2 [21.3]		16	89.5 [19.3]			18	92.8 [23.3]	
**DLCO**	24	38.6 [16.7]		13	37.9 [19.6]			11	39.5 [13.3]	

Legend: BMI, Body Mass Index; CTDs, Connective Tissue Diseases; DLCO; Diffusing Capacity for Carbon Monoxide % predicted; FEV1/FVC, ratio of Forced Expiratory Volume in the First Second to Forced Vital Capacity; ILD, Interstitial Lung Disease; IPF, Idiopathic Pulmonary Fibrosis; SD, Standard Deviation; TLC; Total Lung Capacity % predicted.

**Table 2:** General admission characteristics.

-	**Total**			**NIPPV prior to IMV**			**Only IMV**		
**Variable**	**n**	**Out of**	**%**	**n**	**Out of**	**%**	**n**	**Out of**	**%**
**All Patients**	54	54	100.0%	27	27	100.0%	27	27	100.0%
**How Patients Presented** ED OSH Transfer Another Provider	22 30 2	54	40.7% 55.6% 3.7%	11 15 1	27	40.7% 55.6% 3.7%	11 15 1	27	40.7% 55.6% 3.7%
**Ventilation Mode** Volume Control Pressure Control SIMV Other	24 2 1 14	41	58.5% 4.9% 2.4% 34.1%	11 1 0 8	20	55.0% 5.0% 0.0% 40.0%	13 1 1 6	21	61.9% 4.8% 4.8% 28.6%
**Re-intubation[s]** 0 1 2+	42 7 1	50	84.0% 14.0% 2.0%	23 3 0	26	88.5% 11.5% 0.0%	19 4 1	24	79.2% 16.7% 4.2%
**Tracheostomy**	10	54	18.5%	5	27	18.5%	5	27	18.5%
**Palliative Care Consultation**	21	43	48.8%	11	21	52.4%	10	22	45.5%
**Consideration for Lung Transplant**	11	51	21.6%	6	25	24.0%	5	26	19.2%

**Group**	**n**	**Mean [SD]**		**n**	**Mean [SD]**		**n**	**Mean [SD]**	
**Initial pH**	54	7.38 [0.1]		27	7.40 [0.1]		27	7.34 [0.1]	
**Initial pO2**	54	128.4 [108.1]		27	116.7 [90.3]		27	140.2 [124.0]	
**Initial pCO2**	54	49.8 [17.2]		27	47.9 [16.6]		27	51.8 [17.8]	
**Initial PaO2/FiO2**	42	224.8 [114.7]		21	231.5 [162.2]		21	218.2 [128.5]	

Legend: ED, Emergency Department; OSH, Outside Hospital; SD, Standard Deviation; SIMV, Synchronized Intermittent Mandatory Ventilation.

**Table 3:** Determinants of in-hospital mortality.

** Variables **	** Total **	** P-value **	** NIPPV prior to IMV **	** Only IMV **	** p-value **
All Patients	N=54 Deaths 34 [63.0%]		N=27 Deaths 17 [63.0%]	N=27 Deaths 17 [63.0%]	1.00
	Deaths/n		Deaths/n	Deaths/n	
**BMI** <26.9 26.9+	13/27 [48.1%] 21/27 [77.8%]	**0.047**	5/12 [41.7%] 12/15 [80.0%]	8/15 [53.3%] 9/12 [75.0%]	0.70 1.00
**Age** <65 65+	11/22 [50.0%] 23/32 [71.9%]	0.15	3/9 [33.3%] 14/18 [77.8%]	8/13 [61.5%] 9/14 [64.3%]	0.39 0.45
**Gender** Female Male	15/24 [62.5%] 19/30 [63.3%]	1.00	9/13 [69.2%] 8/14 [57.1%]	6/11 [54.5%] 11/16 [68.8%]	0.68 0.71
**ILD Subtype** IPF CTDs Sarcoidosis Other	7/8 [87.5%] 12/17 [70.6%] 1/7 [14.3%] 14/22 [63.6%]	**0.027**	1/2 [50.0%] 5/8 [62.5%] 1/4 [25.0%] 10/13 [76.9%]	6/6 [100.0%] 7/9 [77.8%] 0/3 [0.0%] 4/9 [44.4%]	0.25 0.62 1.00 0.19
**ILD Duration** ILD <1 year ILD ≥1 year	19/24 [79.2%] 15/30 [50.0%]	**0.046**	10/11 [90.9%] 7/16 [43.8%]	9/13 [69.2%] 8/14 [57.1%]	0.33 0.72
**Supplemental O2** Prescribed Not prescribed	12/25 [48.0%] 22/29 [75.9%]	**0.049**	8/15 [53.3%] 9/12 [75.0%]	4/10 [40.0%] 13/17[76.5%]	0.69 1.00
**Pulmonary Hypertension** Present Not Present	18/28 [64.3%] 16/26 [61.5%]	1.00	7/14 [50.0%] 10/13 [76.9%]	11/14 [78.6%] 6/13 [46.2%]	0.24 0.23
**History of CTD** Present Not Present	16/27 [59.3%] 18/27 [66.7%]	0.78	8/14 [57.1%] 9/13 [69.2%]	8/13 [61.5%] 9/14 [64.3%]	1.00 1.00
**Cause of ARF** Pneumonia/Sepsis ILD Exacerbation All others	21/33 [63.6%] 7/9 [77.8%] 6/12 [50.0%]	0.45	9/17 [52.9%] 5/6 [83.3%] 3/4 [75.0%]	12/16 [75.0%] 2/3 [66.7%] 3/8 [37.5%]	0.28 1.00 0.55
**SVTs** Sinus Tachycardia All Other SVTs	11/21 [52.4%] 20/25 [80.0%]	0.06	4/7 [57.1%] 12/15 [80.0%]	7/14 [50.0%] 8/10 [80.0%]	1.00 1.00
**Vasopressor** Used Not used	30/44 [68.2%] 2/8 [25.0%]	**0.043**	15/22 [68.2%] 0/3 [0.0%]	15/22 [68.2%] 2/5 [40.0%]	1.00 0.46
**Pulmonary Vasodilator** Used Not Used	15/22 [68.2%] 13/26 [50.0%]	0.25	5/8 [62.5%] 8/15 [53.3%]	10/14 [71.4%] 5/11 [45.5%]	1.00 1.00
**Steroid Use** Pulse/Stress Dose Other Dose	19/22 [86.4%] 10/26 [38.5%]	**0.001**	11/13 [84.6%] 6/14 [42.9%]	8/9 [88.9%] 4/12 [33.3%]	1.00 0.70

Legend: Column 3 represents p-value for the mortality of the total number of patients as a comparison of death rate prior to cohort stratification. Column 6 represents p-values for mortality of patients compared within each strata of both NIPPV prior to IMV and Only IMV.

**Table 4:** Mechanical ventilation settings and in-hospital mortality.

** Variables **	** Total **	** P-value **	** NIPPV prior to IMV **	** Only IMV **	** P-value **
All Patients	N=54 Deaths 34 [63.0%]		N=27 Deaths 17 [63.0%]	N=27 Deaths 17 [63.0%]	1.00
	Deaths/n		Deaths/n	Deaths/n	
**Set Respiratory Rate** <25 25+	9/14 [64.3%] 13/15 [86.7%]	0.21	6/8 [75.0%] 6/7 [85.7%]	3/6 [50.0%] 7/8[87.5%]	0.58 1.00
**Set Tidal Volume** <400 400+	10/14 [71.4%] 9/12 [75.0%]	1.00	4/6 [66.7%] 5/6 [83.3%]	6/8 [75.0%] 4/6 [66.7%]	1.00 1.00
**Set PEEP** <10 10+	15/29 [51.7%] 12/12 [100.0%]	**0.003**	9/16 [56.3%] 4/4 [100.0%]	6/13 [46.2%] 8/8 [100.0%]	0.72 1.00
**Set FiO2** <50 50+	11/22 [50.0%] 16/19 [84.2%]	**0.046**	6/11 [54.5%] 7/9 [77.8%]	5/11 [45.5%] 9/10 [90.0%]	1.00 0.58
**Peak Airway Pressure** <30 30+	8/16 [50.0%] 15/16 [93.8%]	0.18	3/7 [42.9%] 8/11 [72.7%]	5/9 [55.6%] 6/8 [75.0%]	1.00 1.00
**Average Airway Pressure** <15 15+	9/21 [42.9%] 15/16 [93.8%]	**0.002**	5/12 [41.7%] 7/7 [100.0%]	4/9 [44.4%] 8/9 [88.9%]	1.00 1.00
**PaO2/FiO2** <150 150+	13/16 [81.3%] 14/26 [53.8%]	0.10	7/8 [87.5%] 6/13 [46.2%]	6/8 [75.0%] 8/13 [61.5%]	1.00 0.70

Legend: Column 3 represents p-value for the mortality of the total number of patients as a comparison of death rate prior to cohort stratification. Column 6 represents p-values for mortality of patients compared within each strata of both NIPPV prior to IMV and Only IMV.

**Table 5:** Determinants of one-year mortality.

** Variables **	** Total **	** P-value **	** NIPPV prior to IMV **		** Only IMV **	** P-value **
All Patients	N=54 Deaths 44 [81.5%]		N=27 Deaths 23 [85.2%]	N=27 Deaths 21 [77.8%]		0.73
	Deaths/n		Deaths/n	Deaths/n		
**BMI** <26.9 26.9+	20/27 [74.1%] 24/27 [88.9%]	0.29	9/12 [75.0%] 14/15 [93.3%]	11/15 [73.3%] 10/12 [83.3%]		1.00 0.57
**Age** <65 65+	16/22 [72.7%] 28/32 [87.5%]	0.28	5/9 [55.6%] 18/18 [100.0%]	11/13 [84.6%] 10/14 [71.4%]		0.18 0.028
**Gender** Female Male	19/24 [79.2%] 25/30 [83.3%]	0.74	11/13 [84.6%] 12/14 [85.7%]	8/11 [72.7%] 13/16 [81.3%]		0.63 1.00
**ILD Subtype** IPF CTD Sarcoidosis Other	8/8 [100.0%] 13/17 [76.5%] 2/7 [28.6%] 21/22 [95.5%]	**0.001**	2/2 [100.0%] 6/8 [75.0%] 2/4 [50.0%] 13/13 [100.0%]	6/6 [100.0%] 7/9 [77.8%] 0/3 [0.0%] 8/9 [88.9%]		1.00 1.00 0.43 0.41
**ILD Presence** <1 year ≥1 year	22/24 [91.7%] 22/30 [73.3%]	0.16	11/11 [100.0%] 12/16 [75.0%]	11/13 [84.6%] 10/14 [71.4%]		0.48 1.00
**Pulmonary Hypertension** Present Not Present	23/28 [82.1%] 21/26 [80.8%]	1.00	10/14 [71.4%] 13/13 [100.0%]	13/14 [92.9%] 8/13 [61.5%]		0.33 0.039
**History of Connective Tissue Disease** Present Not Present	18/27 [66.7%] 26/27 [96.3%]	**0.011**	10/14 [71.4%] 13/13 [100.0%]	8/13 [61.5%] 13/14[92.9%]		0.69 1.00
**Cause of ARF** Pneumonia/Sepsis ILD Exacerbation All others	28/33 [84.8%] 9/9 [100.0%] 7/12 [58.3%]	0.45	14/17 [82.4%] 6/6 [100.0%] 3/4 [75.0%]	14/16 [87.5%] 3/3 [100.0%] 4/8 [50.0%]		1.00 1.00 0.58
**SVTs** Sinus Tachycardia All Other SVTs	15/21 [71.4%] 24/25 [96.0%]	**0.037**	6/7 [85.7%] 14/15 [93.3%]	9/14 [64.3%] 10/10 [100.0%]		0.61 1.00
**Vasopressor** Used Not used	38/44 [86.4%] 4/8 [50.0%]	**0.035**	19/22 [86.4%] 2/3 [66.7%]	19/22 [86.4%] 2/5 [40.0%]		1.00 1.00
**Pulmonary Vasodilator** Used Not Used	19/22 [86.4%] 19/26 [73.1%]	0.31	6/8 [75.0%] 13/15 [86.7%]	13/14 [92.9%] 6/11 [54.5%]		0.53 0.09
**Steroid Use** Pulse/Stress Dose Other Dose	20/22 [90.9%] 18/26 [69.2%]	0.08	12/13 [92.3%] 11/14 [78.6%]	8/9 [88.9%] 7/12 [58.3%]		1.00 0.40

Legend: Column 3 represents p-value for the mortality of the total number of patients as a comparison of death rate prior to cohort stratification. Column 6 represents p-values for mortality of patients compared within each strata of both NIPPV prior to IMV and Only IMV.

## LIST OF ABBREVIATIONS

ARF: = Acute Respiratory Failure
BMI: = Body Mass Index
CTD: = Connective Tissue Disease
DLCO: = Diffusing Capacity for Carbon Monoxide
ECG: = Electrocardiography
ECMO: = Extracorporeal Membrane Oxygenation
ED: = Emergency Department
EMR: = Electronic Medical Record
FEV1/FVC: = Ratio of Forced Expiratory Volume in the First Second to Forced Vital Capacity
ICD: = International Classification of Diseases
ICU: = Intensive Care Unit
ILD: = Interstitial Lung Disease
ILD-ARF: = Interstitial Lung Disease associated Acute Respiratory Failure
IMV: = Invasive Mechanical Ventilation
IPF: = Idiopathic Pulmonary Fibrosis
IRB: = Institutional Review Board
LTx: = Lung Transplantation
NIPPV: = Noninvasive Positive Pressure Ventilation
OSH: = Outside Hospital
PEEP: = Positive End Expiratory Pressure
PFT: = Pulmonary Function Test
PH: = Pulmonary Hypertension
PPV: = Positive Pressure Ventilation
RHC: = Right Heart Catheterization
SIMV: = Synchronized Intermittent Mandatory Ventilation
sPAP: = Systolic Pulmonary Artery Pressure
SVT: = Supraventricular Tachycardia
TLC: = Total Lung Capacity
VILI: = Ventilator Induced Lung Injury
