# Supplementary material for: Interstitial Lung Disease Associated Acute Respiratory Failure Requiring Invasive Mechanical Ventilation: A Retrospective Analysis
Source: Open Respir Med J. 2020 Dec 18;14:67–77. doi: 10.2174/1874306402014010067 (PMC7774098; doi:10.2174/1874306402014010067)
Supplement: Supplementary file 1 — Supplementary material is available on the publisher’s website along with the published article. [file TORMJ-14-67_Sp.pdf]

The Open Respiratory Medicine Journal

Content list available at: https://openrespiratorymedicinejournal.com

Supplementary Material

CrossMark

Interstitial Lung Disease Associated Acute Respiratory Failure Requiring Invasive Mechanical Ventilation: A Retrospective Analysis

Cyrus A Vahdatpour[1,*], Alexander Pichler[2], Harold I Palevsky[3,4], Michael J Kallan[4,5], Namrata B Patel[4,6] and Paul A Kinniry[4,7]

[1]*Division of Pulmonary, Critical Care, and Sleep Medicine, University of Florida, Gainesville, Florida*
[2]*Department of Internal Medicine, Medical University of Vienna, Vienna, Austria*
[3]*Pulmonary, Allergy, and Critical Care Division, Department of Medicine, Penn Presbyterian Medical Center, Philadelphia, Pennsylvania*
[4]*Perelman School of Medicine, University of Pennsylvania, Philadelphia, Pennsylvania*
[5]*Department of Biostatistics, Epidemiology, and Informatics, Perelman School of Medicine, University of Pennsylvania, Philadelphia, Pennsylvania*
[6]*Pulmonary, Allergy, and Critical Care Division, Department of Medicine, Hospital of the University of Pennsylvania, Philadelphia, Pennsylvania*
[7]*Pulmonary, Allergy, and Critical Care Division, Department of Medicine, Pennsylvania Hospital, Philadelphia, Pennsylvania*

Article History	Received: July 15, 2020	Revised: September 22, 2020	Accepted: September 30, 2020

SUPPLEMENTARY TABLE AND FIGURE

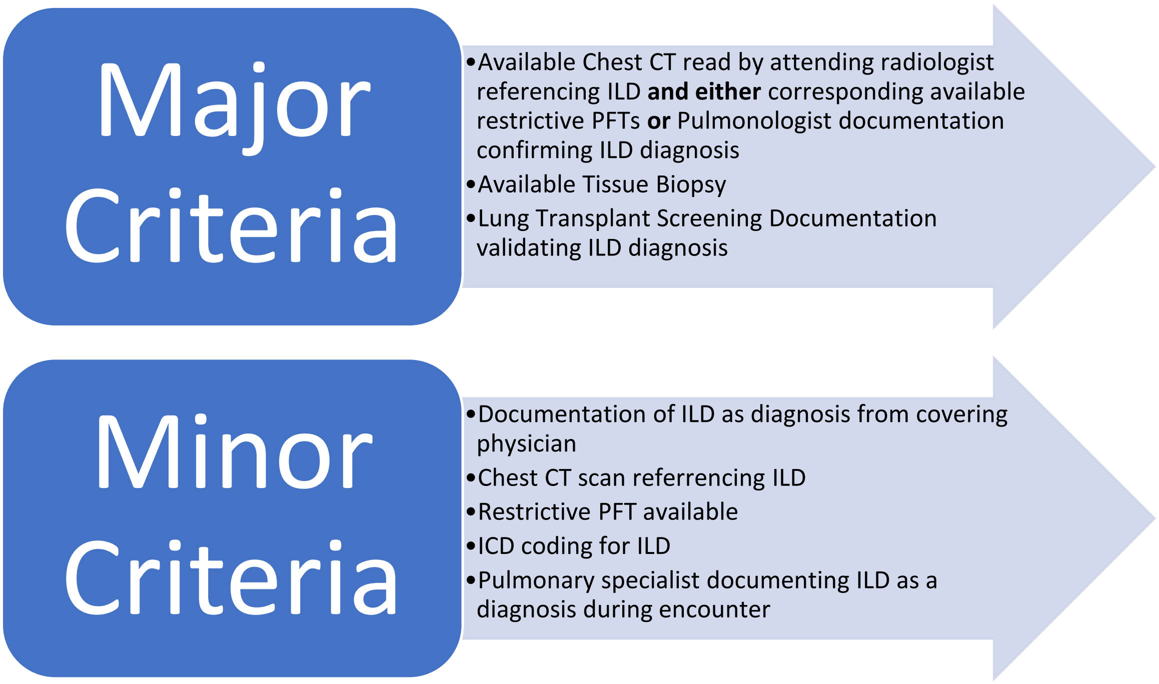

Supplementary Fig. 1. Major and Minor Criteria for Inclusion.

Supplementary Table 1. Determinants of In-Hospital Mortality.

Variables	Total	p-value	NIPPV prior to IMV	IMV only	p-value
All Patients	N=54<br>Deaths 34<br>(63.0%)		N=27<br>Deaths 17<br>(63.0%)	N=27<br>Deaths 17<br>(63.0%)	1.00
	Deaths/n		Deaths/n	Deaths/n	
**Diastolic Dysfunction**<br>Present<br>Not present	 15/23 (65.2%)<br>15/25 (60.0%)	0.77	 6/9 (66.7%)<br>9/14 (64.3%)	 6/9 (66.7%)<br>9/14 (64.3%)	 1.00<br>0.70
**Pulmonary Arterial Systolic Pressure**<br><45<br>45+	 11/17 (64.7%)<br>14/22 (63.6%)	1.00	 8/11 (72.7%)<br>5/10 (50.0%)	 3/6 (50.0%)<br>9/12 (75.0%)	 0.60<br>0.38
**Peak Tricuspid Regurgitant Velocity**<br><2.9m/s<br>+2.9m/s	 9/16 (56.3%)<br>14/19 (73.7%)	0.31	 4/8 (50.0%)<br>6/9 (66.7)	 5/8 (62.5%)<br>8/10 (80.0%)	 1.00<br>0.63
**Tricuspid Annular Plane Systolic Excursion**<br><1.8<br>+1.8	 7/9 (77.8%)<br>11/21 (52.4%)	0.25	 5/5 (100.0%)<br>4/8 (50.0%)	 2/4 (50.0%)<br>7/13 (53.8%)	 0.17<br>1.00
**Right Atrial Pressure**<br><6<br>6+	 9/15 (60.0%)<br>22/33 (66.7%)	0.75	 5/7 (71.4%)<br>10/17 (58.8%)	 4/8 (50.0%)<br>12/16 (75.0%)	 0.61<br>0.46
**Days in ICU**<br>0-14<br>15+	 18/27 (66.7%)<br>16/27 (59.3%)	0.78	 10/13 (76.9%)<br>7/14 (50.0%)	 8/14 (57.1%)<br>9/13 (69.2%)	 0.42<br>0.44
**Days in Hospital**<br>0-20<br>21+	 22/27 (81.5%)<br>12/27 (44.4%)	**0.010**	 12/14 (85.7%)<br>5/13 (38.5%)	 10/13 (76.9%)<br>7/14 (50.0%)	 0.65<br>0.70

Legend: Column 3 represents p-value for the mortality of the total number of patients as a comparison of death rate prior to cohort stratification. Column 6 represents p-values for mortality of patients compared within each strata of both NIPPV prior to IMV and IMV only.

Supplementary Table 2. Determinants of One-Year Mortality.

Variables	Total	p-value	NIPPV prior to IMV	IMV only	p-value
All Patients	N=54<br>Deaths 34<br>(63.0%)		N=27<br>Deaths 17<br>(63.0%)	N=27<br>Deaths 17<br>(63.0%)	1.00
	Deaths/n		Deaths/n	Deaths/n	
**Diastolic Dysfunction**<br>Present<br>Not present	 20/23 (87.0%)<br>18/25 (72.0%)	0.29	 8/9 (88.9%)<br>11/14 (78.6%)	 12/14 (85.7%)<br>7/11 (63.6%)	 1.00<br>0.66
**Pulmonary Arterial Systolic Pressure**<br><45<br>45+	 14/17 (82.4%)<br>19/22 (86.4%)	1.00	 10/11 (90.9%)<br>8/10 (80.0%)	 4/6 (66.7%)<br>11/12 (91.7%)	 0.51<br>0.57
**Peak Tricuspid Regurgitant Velocity**<br><2.9m/s<br>+2.9m/s	 13/16 (81.3%)<br>18/19 (94.7%)	0.31	 7/8 (87.5%)<br>8/9 (88.9%)	 6/8 (75.0%)<br>10/10 (100.0%)	 1.00<br>0.47
**Tricuspid Annular Plane Systolic Excursion**<br><1.8<br>+1.8	 7/9 (77.8%)<br>17/21 (81.0%)	1.00	 55/5 (100.0%)<br>6/8 (75.0%)	 2/4 (50.0%)<br>11/13 (84.6%)	 0.17<br>0.62
**Right Atrial Pressure**<br><6<br>6+	 12/15 (80.0%)<br>27/33 (81.8%)	1.00	 6/7 (85.7%)<br>14/17 (82.4%)	 6/8 (75.0%)<br>13/16 (81.3%)	 1.00<br>1.00
**Days in ICU**<br>0-14<br>15+	 20/27 (74.1%)<br>24/27 (88.9%)	0.29	 11/13 (84.6%)<br>12/14 (85.7%)	 9/14 (64.3%)<br>12/13 (92.3%)	 0.38<br>1.00
**Days in Hospital**<br>0-20<br>21+	 22/27 (81.5%)<br>22/27 (81.5%)	**0.010**	 12/14 (85.7%)<br>11/13 (84.6%)	 10/13 (76.9%)<br>11/14 (78.6%)	 0.65<br>1.00

Legend: Column 3 represents p-value for the mortality of the total number of patients as a comparison of death rate prior to cohort stratification. Column 6 represents p-values for mortality of patients compared within each strata of both NIPPV prior to IMV and IMV only.

Supplementary Table 3. In-hospital mortality of Patients with and without PH

Variables	Total	p-value	PH	No PH	p-value
All Patients	N=54<br>Deaths 34<br>(63.0%)		N=28<br>Deaths 18<br>(64.3%)	N=26<br>Deaths 16<br>(61.5%)	1.00
	Deaths/n		Deaths/n	Deaths/n	
**Age**		0.15			
<65	11/22 (50.0%)		3/10 (30.0%)	8/12 (66.7%)	0.20
65+	23/32 (71.9%)		15/18 (83.3%)	9/14 (57.1%)	0.13
**Gender**		1.00			
Female	15/24 (62.5%)		7/13 (53.8%)	8/11 (72.7%)	0.42
Male	19/30 (63.3%)		11/15 (73.3%)	8/15 (53.3%)	0.45
**ILD Subtype**		**0.027**			
IPF	7/8 (87.5%)		6/7 (85.7%)	1/1 (100.0%)	1.00
CTDs	12/17 (70.6%)		3/6 (50.0%)	9/11 (81.8%)	0.28
Sarcoidosis	1/7 (14.3%)		0/3 (0.0%)	1/4 (25.0%)	1.00
Other	14/22 (63.6%)		9/12 (75.0%)	5/10 (44.4%)	0.38
**ILD Duration**		**0.046**			
ILD <1 year	19/24 (79.2%)		9/13 (69.2%)	10/11 (90.9%)	0.33
ILD ≥1 year	15/30 (50.0%)		9/15 (60.0%)	6/15 (40.0%)	0.47
**Peak Tricuspid Regurgitation**		0.31			
<2.9	9/16 (56.3%)		3/5 (60.0%)	6/11 (54.5%)	1.00
2.9+	14/19 (73.7%)		12/16 (75.0%)	2/3 (66.7)	1.00
**Right Atrial Pressure**		0.75			
<6	9/15 (60.0%)		2/4 (50.0%)	7/11 (63.6%)	1.00
6+	22/33 (66.7)		14/22 (63.6%)	8/11 (72.7%)	0.71
**Supplemental O2**		**0.049**			
Prescribed	12/25 (48.0%)		7/14 (50.0%)	5/11 (45.5%)	1.00
Not prescribed	22/29 (75.9%)		11/14 (78.6.0%)	11/15 (73.3%)	1.00
**Vasopressor**		**0.043**			
Used	30/44 (68.2%)		15/24 (62.5%)	15/20 (75.0%)	0.52
Not used	2/8 (25.0%)		2/3 (66.7%)	0/5 (0.0%)	0.11
**Steroid Use**		**0.001**			
Pulse/Stress Dose	19/22 (86.4%)		9/11 (81.8%)	10/11 (90.9%)	1.00
Other Dose	10/26 (38.5%)		5/13 (38.5%)	5/13 (38.5%)	1.00

Legend: Column 3 represents p-value for the mortality of the total number of patients as a comparison of death rate prior to cohort stratification. Column 6 represents p-values for mortality of patients compared within each strata of both NIPPV prior to IMV and IMV only.

Supplementary Table 4. General ICU and Ventilation Data for All Included Patients.

Variables	n	Mean (sd)
Days in ICU	54	17.94 (16.73)
Days in Hospital	54	22.17 (17.01)
Days on IMV	54	13.61 (15.40)
Days on PPV	54	14.33 (15.23)
Set Respiratory Rate	29	24.69 (6.86)
Tidal Volume (ml)	26	375.38 (71.85)
PEEP	41	7.67 (3.04)
FiO2	41	55.12 (22.29)
Peak Airway Pressure	35	30.31 (9.83)
Average Airway Pressure	37	14.57 (5.14)
Pa/FiO2	42	224.82 (144.70)

Supplementary Table 5. Subtypes of ILD.

ILD Subtype	Number (Total 54)	Percent of Cohort
IPF	8	14.8%
Sarcoidosis	7	13.0%
Scleroderma	7	13.0%

(Supplementary Table 5) contd.....

ILD Subtype	Number (Total 54)	Percent of Cohort
CTD associated	10	18.5%
NSIP	3	5.6%
COP	1	1.9%
Drug related	2	3.7%
Unclear	16	29.6%

Supplementary Table 6. Causes of ARF.

Etiology	Number (Total 54)	Percent of Cohort
Pneumonia related Sepsis	33	61.1%
ILD exacerbation	9	16.6%
Non-pneumonia related Sepsis	2	3.7%
Congestive Heart Failure	1	1.9%
Pneumothorax	2	3.7%
Pulmonary Emboli	1	1.9%
Unclear	6	11.1%

© 2020 Vahdatpour *et al*.

This is an open access article distributed under the terms of the Creative Commons Attribution 4.0 International Public License (CC-BY 4.0), a copy of which is available at: https://creativecommons.org/licenses/by/4.0/legalcode. This license permits unrestricted use, distribution, and reproduction in any medium, provided the original author and source are credited.
